# 3,4-Dimeth­oxy-*N*-(4-nitro­benzyl­idene)­aniline

**DOI:** 10.1107/S1600536808032042

**Published:** 2008-10-09

**Authors:** Mehmet Akkurt, Aliasghar Jarrahpour, Malihe Aye, Mustafa Gençaslan, Orhan Büyükgüngör

**Affiliations:** a Department of Physics, Faculty of Arts and Sciences, Erciyes University, 38039 Kayseri, Turkey; b Department of Chemistry, College of Sciences, Shiraz University, 71454 Shiraz, Iran; c Department of Physics, Faculty of Arts and Sciences, Ondokuz Mayıs University, 55139 Samsun, Turkey

## Abstract

In the title mol­ecule, C_15_H_14_N_2_O_4_, the dihedral angle between the two benzene rings is 29.52 (8)°. The nitro and two meth­oxy substituents are almost coplanar with their respective benzene rings. The crystal structure is stabilized by inter­molecular C—H⋯O inter­actions.

## Related literature

For general background, see: Bey & Vevert (1977 ▶); Bezas & Zervas (1961 ▶); Fleet & Fleming (1969 ▶); Lucas *et al.* (1960 ▶); Macho *et al.* (2004 ▶). For a related structure, see: Akkurt *et al.* (2005 ▶).
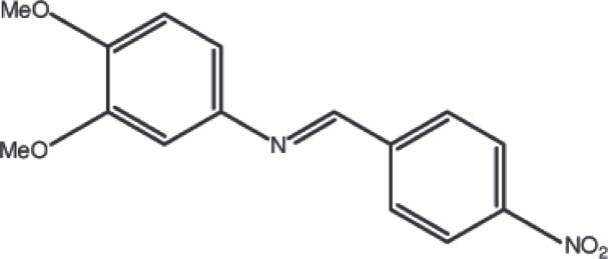


## Experimental

### 

#### Crystal data

C_15_H_14_N_2_O_4_
*M* *_r_* = 286.28
Monoclinic, 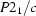

*a* = 7.9536 (4) Å
*b* = 8.2258 (3) Å
*c* = 21.3418 (10) Å
β = 96.125 (4)°
*V* = 1388.31 (11) Å^3^
*Z* = 4
Mo *K*α radiation
μ = 0.10 mm^−1^
*T* = 296 K
0.25 × 0.21 × 0.18 mm

#### Data collection

Stoe IPDS II diffractometer
Absorption correction: integration (*X-RED32*; Stoe & Cie, 2002 ▶) *T*_min_ = 0.975, *T*_max_ = 0.982
14598 measured reflections
2880 independent reflections
1797 reflections with *I* > 2σ(*I*)
*R*_int_ = 0.038

#### Refinement

*R*[*F* ^2^ > 2σ(*F* ^2^)] = 0.040
*wR*(*F* ^2^) = 0.120
*S* = 0.83
2880 reflections
191 parameters
H-atom parameters constrained
Δρ_max_ = 0.13 e Å^−3^
Δρ_min_ = −0.12 e Å^−3^

### 

Data collection: *X-AREA* (Stoe & Cie, 2002 ▶); cell refinement: *X-AREA*; data reduction: *X-RED32* (Stoe & Cie, 2002 ▶); program(s) used to solve structure: *SHELXS97* (Sheldrick, 2008 ▶); program(s) used to refine structure: *SHELXL97* (Sheldrick, 2008 ▶); molecular graphics: *ORTEP-3* (Farrugia, 1997 ▶); software used to prepare material for publication: *WinGX* (Farrugia, 1999 ▶).

## Supplementary Material

Crystal structure: contains datablocks global, I. DOI: 10.1107/S1600536808032042/tk2312sup1.cif

Structure factors: contains datablocks I. DOI: 10.1107/S1600536808032042/tk2312Isup2.hkl

Additional supplementary materials:  crystallographic information; 3D view; checkCIF report

## Figures and Tables

**Table 1:** Hydrogen-bond geometry (Å, °)

*D*—H⋯*A*	*D*—H	H⋯*A*	*D*⋯*A*	*D*—H⋯*A*
C8—H8*C*⋯O1^i^	0.96	2.55	3.255 (3)	130
C8—H8*C*⋯O4^ii^	0.96	2.56	3.405 (3)	147
C14—H14⋯O2^iii^	0.93	2.56	3.246 (2)	131

Symmetry codes: (i) 
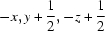
; (ii) 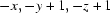; (iii) 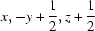.

**Fig. 1.**
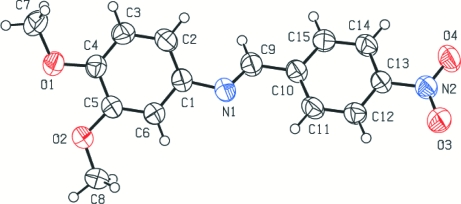
View of (I) showing with the atom-numbering scheme and 50% probability displacement ellipsoids

**Fig. 2.**
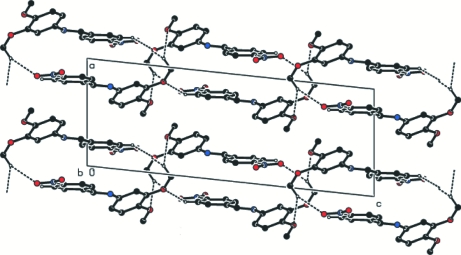
View down the b axis of the packing and C—H···O contacts in the unit cell of (I). H atoms not involved in C—H···O contacts (dashed lines) have been omitted for clarity.

## supplementary crystallographic information

### Comment

Schiff bases belong to a widely used group of organic intermediates which are important for production of certain chemicals, such as pharmaceuticals and rubber additives (Macho *et al.*, 2004), and as amino protective groups in organic synthesis (Bey & Vevert, 1977; Lucas *et al.*, 1960; Fleet & Fleming, 1969; Bezas & Zervas, 1961). As we are interested in Schiff bases, we report here the crystal structure of the title compound, (I).

In (I), Fig. 1, the dihedral angle between the two benzene rings (C1–C6) and (C10–C15) is 29.52 (8)° and the C1—N1—C9—C10 torsion angle is 176.12 (15)°. The nitro and two methoxy substituents are coplanar with their respective benzene rings.

The crystal structure of (I) is stabilized by intermolecular C—H···O interactions, Fig. 2 and Table 1.

### Experimental

A mixture of 3,4-dimethoxyaniline (3 mmol) and 4-nitrobenzaldehyde (3 mmol) was refluxed in EtOH for 4 h. After cooling the solution, the formed precipitate was filtered off and washed with ethanol to give pure Schiff base as an orange solid in an 89% yield; m. pt. = 429–431 K. IR (KBr, cm^-1^): 1600.3 (C?N). ^1^H NMR (CDCl_3_) δ 3.92, 3.94 (2 x OCH_3_, s, 6H), 6.92–8.66 (ArH, m, 7H), 8.90 (HC?N, s, 1H). ^13^C NMR (CDCl_3_) δ 55.97, 56.12 (2 OCH_3_), 105.63–149.50 (C?C aromatic carbons), 155.03 (C?N).

### Refinement

After checking for their presence in the Fourier map, all hydrogen atoms were placed in calculated positions and allowed to ride on their parent atoms with the C—H = 0.93 Å (aromatic) and C—H = 0.96 Å (methy) with *U*_iso_(H) = 1.2*U*_eq_(C_aromatic_) and 1.5*U*_eq_(C_methyl_).

### Crystal data

**Table:**

C_15_H_14_N_2_O_4_	*F*(000) = 600
*M**_r_* = 286.28	*D*_x_ = 1.370 Mg m^−^^3^
Monoclinic, *P*2_1_/*c*	Mo *K*α radiation, λ = 0.71073 Å
Hall symbol: -P 2ybc	Cell parameters from 11667 reflections
*a* = 7.9536 (4) Å	θ = 1.9–27.3°
*b* = 8.2258 (3) Å	µ = 0.10 mm^−^^1^
*c* = 21.3418 (10) Å	*T* = 296 K
β = 96.125 (4)°	Prism, brown
*V* = 1388.31 (11) Å^3^	0.25 × 0.21 × 0.18 mm
*Z* = 4	

### Data collection

**Table:**

Stoe IPDS II diffractometer	2880 independent reflections
Radiation source: sealed X-ray tube, 12 x 0.4 mm long-fine focus	1797 reflections with *I* > 2σ(*I*)
plane graphite	*R*_int_ = 0.038
Detector resolution: 6.67 pixels mm^-1^	θ_max_ = 26.5°, θ_min_ = 1.9°
ω scans	*h* = −9→9
Absorption correction: integration (X-RED32; Stoe & Cie, 2002)	*k* = −10→10
*T*_min_ = 0.975, *T*_max_ = 0.982	*l* = −26→26
14598 measured reflections	

### Refinement

**Table:**

Refinement on *F*^2^	Secondary atom site location: difference Fourier map
Least-squares matrix: full	Hydrogen site location: inferred from neighbouring sites
*R*[*F*^2^ > 2σ(*F*^2^)] = 0.040	H-atom parameters constrained
*wR*(*F*^2^) = 0.120	*w* = 1/[σ^2^(*F*_o_^2^) + (0.0824*P*)^2^] where *P* = (*F*_o_^2^ + 2*F*_c_^2^)/3
*S* = 0.83	(Δ/σ)_max_ < 0.001
2880 reflections	Δρ_max_ = 0.13 e Å^−^^3^
191 parameters	Δρ_min_ = −0.12 e Å^−^^3^
0 restraints	Extinction correction: SHELXL97 (Sheldrick, 2008), Fc*=KFc[1+0.001Fc^2^λ^3^/sin(2θ)]^-1/4^
Primary atom site location: structure-invariant direct methods	Extinction coefficient: 0.0071 (16)

### Special details

**Table:**

Geometry. Bond distances, angles *etc*. have been calculated using the rounded fractional coordinates. All su's are estimated from the variances of the (full) variance-covariance matrix. The cell e.s.d.'s are taken into account in the estimation of distances, angles and torsion angles
Refinement. Refinement on *F*^2^ for ALL reflections except those flagged by the user for potential systematic errors. Weighted *R*-factors *wR* and all goodnesses of fit *S* are based on *F*^2^, conventional *R*-factors *R* are based on *F*, with *F* set to zero for negative *F*^2^. The observed criterion of *F*^2^ > σ(*F*^2^) is used only for calculating -*R*-factor-obs *etc*. and is not relevant to the choice of reflections for refinement. *R*-factors based on *F*^2^ are statistically about twice as large as those based on *F*, and *R*-factors based on ALL data will be even larger.

### Fractional atomic coordinates and isotropic or equivalent isotropic displacement parameters (Å^2^)

**Table:**

	*x*	*y*	*z*	*U*_iso_*/*U*_eq_	
O1	0.34730 (16)	−0.17529 (15)	0.28084 (6)	0.0633 (4)	
O2	0.14131 (16)	0.04285 (15)	0.23416 (6)	0.0624 (4)	
O3	0.1545 (2)	1.09341 (17)	0.58860 (7)	0.0859 (6)	
O4	0.2133 (2)	0.96182 (16)	0.67456 (6)	0.0775 (6)	
N1	0.2395 (2)	0.38177 (18)	0.41916 (7)	0.0580 (5)	
N2	0.1851 (2)	0.96644 (18)	0.61754 (7)	0.0578 (5)	
C1	0.2679 (2)	0.2324 (2)	0.38839 (8)	0.0524 (6)	
C2	0.3732 (2)	0.1113 (2)	0.41434 (8)	0.0581 (6)	
C3	0.4020 (2)	−0.0275 (2)	0.37992 (9)	0.0575 (6)	
C4	0.3240 (2)	−0.0470 (2)	0.31966 (8)	0.0509 (5)	
C5	0.2137 (2)	0.0741 (2)	0.29350 (8)	0.0503 (6)	
C6	0.1891 (2)	0.2128 (2)	0.32730 (8)	0.0536 (6)	
C7	0.4578 (3)	−0.3022 (3)	0.30482 (11)	0.0780 (8)	
C8	0.0173 (3)	0.1542 (2)	0.20751 (9)	0.0637 (7)	
C9	0.2415 (2)	0.3850 (2)	0.47827 (9)	0.0571 (6)	
C10	0.2237 (2)	0.5361 (2)	0.51351 (8)	0.0520 (6)	
C11	0.1908 (2)	0.6842 (2)	0.48291 (8)	0.0574 (6)	
C12	0.1756 (2)	0.8243 (2)	0.51682 (8)	0.0566 (6)	
C13	0.1945 (2)	0.8156 (2)	0.58166 (8)	0.0492 (5)	
C14	0.2255 (2)	0.6721 (2)	0.61381 (8)	0.0560 (6)	
C15	0.2377 (2)	0.5328 (2)	0.57890 (8)	0.0588 (6)	
H2	0.42540	0.12260	0.45520	0.0700*	
H3	0.47450	−0.10770	0.39770	0.0690*	
H6	0.11900	0.29450	0.30920	0.0640*	
H7A	0.46300	−0.38430	0.27310	0.1170*	
H7B	0.41610	−0.34920	0.34130	0.1170*	
H7C	0.56890	−0.25870	0.31620	0.1170*	
H8A	−0.02420	0.11930	0.16580	0.0960*	
H8B	0.06680	0.26030	0.20550	0.0960*	
H8C	−0.07470	0.15840	0.23320	0.0960*	
H9	0.25480	0.28770	0.50050	0.0690*	
H11	0.17910	0.68810	0.43910	0.0690*	
H12	0.15290	0.92300	0.49640	0.0680*	
H14	0.23770	0.66920	0.65760	0.0670*	
H15	0.25570	0.43380	0.59960	0.0710*	

### Atomic displacement parameters (Å^2^)

**Table:**

	*U*^11^	*U*^22^	*U*^33^	*U*^12^	*U*^13^	*U*^23^
O1	0.0634 (8)	0.0591 (7)	0.0660 (8)	0.0120 (6)	0.0008 (6)	−0.0126 (6)
O2	0.0720 (8)	0.0637 (8)	0.0490 (7)	0.0131 (6)	−0.0053 (6)	−0.0096 (6)
O3	0.1359 (14)	0.0530 (8)	0.0664 (9)	0.0118 (8)	−0.0004 (9)	0.0023 (7)
O4	0.1171 (12)	0.0690 (9)	0.0466 (8)	−0.0019 (8)	0.0094 (8)	−0.0067 (7)
N1	0.0677 (10)	0.0568 (9)	0.0489 (9)	−0.0007 (7)	0.0037 (7)	−0.0073 (7)
N2	0.0698 (10)	0.0533 (9)	0.0501 (9)	−0.0024 (7)	0.0059 (7)	−0.0002 (7)
C1	0.0570 (10)	0.0523 (10)	0.0483 (9)	−0.0023 (8)	0.0075 (8)	−0.0046 (8)
C2	0.0580 (10)	0.0687 (12)	0.0467 (10)	0.0004 (9)	0.0009 (8)	−0.0034 (9)
C3	0.0532 (10)	0.0606 (11)	0.0581 (11)	0.0081 (8)	0.0033 (8)	0.0016 (9)
C4	0.0490 (9)	0.0505 (9)	0.0538 (10)	0.0010 (8)	0.0081 (8)	−0.0052 (8)
C5	0.0511 (10)	0.0546 (10)	0.0451 (9)	−0.0018 (8)	0.0045 (7)	−0.0038 (8)
C6	0.0606 (11)	0.0525 (10)	0.0475 (9)	0.0049 (8)	0.0055 (8)	−0.0008 (8)
C7	0.0742 (14)	0.0677 (13)	0.0899 (16)	0.0252 (11)	−0.0007 (12)	−0.0115 (11)
C8	0.0767 (13)	0.0642 (12)	0.0482 (10)	0.0096 (9)	−0.0024 (9)	0.0049 (9)
C9	0.0669 (11)	0.0538 (10)	0.0508 (10)	−0.0017 (9)	0.0076 (8)	−0.0026 (8)
C10	0.0577 (10)	0.0530 (10)	0.0455 (9)	−0.0036 (8)	0.0063 (8)	−0.0023 (8)
C11	0.0716 (12)	0.0605 (11)	0.0393 (9)	−0.0030 (9)	0.0021 (8)	0.0017 (8)
C12	0.0707 (12)	0.0508 (10)	0.0472 (10)	−0.0016 (8)	0.0019 (8)	0.0042 (8)
C13	0.0545 (10)	0.0501 (9)	0.0430 (9)	−0.0058 (7)	0.0053 (7)	−0.0017 (7)
C14	0.0718 (12)	0.0566 (10)	0.0399 (9)	−0.0004 (9)	0.0073 (8)	0.0030 (8)
C15	0.0786 (13)	0.0503 (10)	0.0478 (10)	0.0033 (9)	0.0081 (9)	0.0049 (8)

### Geometric parameters (Å, °)

**Table:**

O1—C4	1.366 (2)	C11—C12	1.373 (2)
O1—C7	1.424 (3)	C12—C13	1.378 (2)
O2—C5	1.359 (2)	C13—C14	1.374 (2)
O2—C8	1.420 (2)	C14—C15	1.376 (2)
O3—N2	1.225 (2)	C2—H2	0.9300
O4—N2	1.214 (2)	C3—H3	0.9300
N1—C1	1.423 (2)	C6—H6	0.9300
N1—C9	1.260 (2)	C7—H7A	0.9600
N2—C13	1.464 (2)	C7—H7B	0.9600
C1—C2	1.379 (2)	C7—H7C	0.9600
C1—C6	1.394 (2)	C8—H8A	0.9600
C2—C3	1.390 (2)	C8—H8B	0.9600
C3—C4	1.376 (3)	C8—H8C	0.9600
C4—C5	1.403 (2)	C9—H9	0.9300
C5—C6	1.375 (2)	C11—H11	0.9300
C9—C10	1.467 (2)	C12—H12	0.9300
C10—C11	1.394 (2)	C14—H14	0.9300
C10—C15	1.388 (2)	C15—H15	0.9300
			
O1···O2	2.5589 (18)	H2···H9	2.2100
O1···C8^i^	3.255 (3)	H3···C7	2.5400
O2···C14^ii^	3.246 (2)	H3···H7B	2.3400
O2···O1	2.5589 (18)	H3···H7C	2.3300
O3···C12^iii^	3.340 (2)	H6···C8	2.5100
O4···C8^iv^	3.405 (3)	H6···H8B	2.2300
O1···H8C^i^	2.5500	H6···H8C	2.3900
O1···H14^ii^	2.6800	H7A···C4^xiii^	3.0500
O2···H14^ii^	2.5600	H7A···C5^xiii^	3.0900
O3···H12	2.4100	H7A···O4^ii^	2.8100
O3···H15^v^	2.9200	H7B···C3	2.7800
O3···H12^iii^	2.8900	H7B···H3	2.3400
O3···H9^v^	2.6500	H7C···C3	2.7600
O4···H14	2.4400	H7C···H3	2.3300
O4···H8C^iv^	2.5600	H7C···H15^xii^	2.5900
O4···H7A^vi^	2.8100	H8A···H11^i^	2.5000
O4···H8B^vii^	2.6800	H8B···C6	2.7000
N2···C3^viii^	3.317 (2)	H8B···H6	2.2300
N1···H11	2.6100	H8B···O4^xiv^	2.6800
C2···C13^viii^	3.483 (2)	H8C···C6	2.7800
C3···N2^viii^	3.317 (2)	H8C···H6	2.3900
C8···O4^iv^	3.405 (3)	H8C···O1^ix^	2.5500
C8···O1^ix^	3.255 (3)	H8C···C7^ix^	3.0900
C10···C10^iv^	3.593 (2)	H8C···O4^iv^	2.5600
C12···O3^iii^	3.340 (2)	H9···O3^x^	2.6500
C13···C2^viii^	3.483 (2)	H9···C2	2.6000
C14···O2^vi^	3.246 (2)	H9···H2	2.2100
C2···H9	2.6000	H9···H15	2.4300
C2···H12^x^	3.0300	H11···N1	2.6100
C3···H7C	2.7600	H11···H8A^ix^	2.5000
C3···H7B	2.7800	H12···O3	2.4100
C4···H7A^xi^	3.0500	H12···C2^v^	3.0300
C5···H7A^xi^	3.0900	H12···O3^iii^	2.8900
C6···H8C	2.7800	H14···O4	2.4400
C6···H8B	2.7000	H14···O1^vi^	2.6800
C7···H15^xii^	3.0900	H14···O2^vi^	2.5600
C7···H3	2.5400	H15···O3^x^	2.9200
C7···H8C^i^	3.0900	H15···H9	2.4300
C8···H6	2.5100	H15···C7^xii^	3.0900
C9···H2	2.6800	H15···H7C^xii^	2.5900
H2···C9	2.6800		
			
C4—O1—C7	117.82 (15)	C1—C2—H2	120.00
C5—O2—C8	117.30 (13)	C3—C2—H2	120.00
C1—N1—C9	119.65 (15)	C2—C3—H3	120.00
O3—N2—O4	122.53 (15)	C4—C3—H3	120.00
O3—N2—C13	118.52 (15)	C1—C6—H6	120.00
O4—N2—C13	118.90 (14)	C5—C6—H6	120.00
N1—C1—C2	123.93 (15)	O1—C7—H7A	109.00
N1—C1—C6	116.99 (15)	O1—C7—H7B	109.00
C2—C1—C6	119.02 (15)	O1—C7—H7C	109.00
C1—C2—C3	120.56 (16)	H7A—C7—H7B	109.00
C2—C3—C4	120.42 (15)	H7A—C7—H7C	109.00
O1—C4—C3	125.44 (15)	H7B—C7—H7C	109.00
O1—C4—C5	115.25 (15)	O2—C8—H8A	110.00
C3—C4—C5	119.30 (15)	O2—C8—H8B	109.00
O2—C5—C4	114.92 (14)	O2—C8—H8C	109.00
O2—C5—C6	125.18 (15)	H8A—C8—H8B	109.00
C4—C5—C6	119.88 (15)	H8A—C8—H8C	109.00
C1—C6—C5	120.77 (15)	H8B—C8—H8C	109.00
N1—C9—C10	122.64 (16)	N1—C9—H9	119.00
C9—C10—C11	121.59 (16)	C10—C9—H9	119.00
C9—C10—C15	119.79 (15)	C10—C11—H11	120.00
C11—C10—C15	118.62 (15)	C12—C11—H11	120.00
C10—C11—C12	120.62 (16)	C11—C12—H12	121.00
C11—C12—C13	118.65 (15)	C13—C12—H12	121.00
N2—C13—C12	118.37 (15)	C13—C14—H14	121.00
N2—C13—C14	118.90 (15)	C15—C14—H14	121.00
C12—C13—C14	122.72 (16)	C10—C15—H15	119.00
C13—C14—C15	117.66 (16)	C14—C15—H15	119.00
C10—C15—C14	121.69 (15)		
			
C7—O1—C4—C5	−179.92 (16)	C3—C4—C5—O2	−179.20 (15)
C7—O1—C4—C3	1.8 (2)	O1—C4—C5—C6	−176.25 (15)
C8—O2—C5—C6	−6.9 (2)	O1—C4—C5—O2	2.4 (2)
C8—O2—C5—C4	174.56 (15)	C3—C4—C5—C6	2.2 (2)
C1—N1—C9—C10	176.12 (15)	C4—C5—C6—C1	−2.3 (2)
C9—N1—C1—C2	−33.0 (3)	O2—C5—C6—C1	179.20 (15)
C9—N1—C1—C6	149.95 (17)	N1—C9—C10—C11	4.9 (3)
O3—N2—C13—C12	2.7 (2)	N1—C9—C10—C15	−175.92 (16)
O4—N2—C13—C12	−174.90 (16)	C9—C10—C11—C12	−179.62 (15)
O3—N2—C13—C14	−178.74 (16)	C15—C10—C11—C12	1.2 (2)
O4—N2—C13—C14	3.6 (2)	C9—C10—C15—C14	178.57 (15)
N1—C1—C6—C5	178.12 (15)	C11—C10—C15—C14	−2.2 (2)
C2—C1—C6—C5	0.9 (2)	C10—C11—C12—C13	0.4 (2)
N1—C1—C2—C3	−176.33 (16)	C11—C12—C13—N2	177.51 (15)
C6—C1—C2—C3	0.7 (2)	C11—C12—C13—C14	−1.0 (2)
C1—C2—C3—C4	−0.8 (3)	N2—C13—C14—C15	−178.48 (15)
C2—C3—C4—O1	177.65 (15)	C12—C13—C14—C15	0.0 (2)
C2—C3—C4—C5	−0.6 (2)	C13—C14—C15—C10	1.6 (2)

Symmetry codes: (i) −*x*, *y*−1/2, −*z*+1/2; (ii) *x*, −*y*+1/2, *z*−1/2; (iii) −*x*, −*y*+2, −*z*+1; (iv) −*x*, −*y*+1, −*z*+1; (v) *x*, *y*+1, *z*; (vi) *x*, −*y*+1/2, *z*+1/2; (vii) *x*, −*y*+3/2, *z*+1/2; (viii) −*x*+1, −*y*+1, −*z*+1; (ix) −*x*, *y*+1/2, −*z*+1/2; (x) *x*, *y*−1, *z*; (xi) −*x*+1, *y*+1/2, −*z*+1/2; (xii) −*x*+1, −*y*, −*z*+1; (xiii) −*x*+1, *y*−1/2, −*z*+1/2; (xiv) *x*, −*y*+3/2, *z*−1/2.

### Hydrogen-bond geometry (Å, °)

**Table:**

*D*—H···*A*	*D*—H	H···*A*	*D*···*A*	*D*—H···*A*
C8—H8C···O1^ix^	0.96	2.55	3.255 (3)	130
C8—H8C···O4^iv^	0.96	2.56	3.405 (3)	147
C14—H14···O2^vi^	0.93	2.56	3.246 (2)	131

Symmetry codes: (ix) −*x*, *y*+1/2, −*z*+1/2; (iv) −*x*, −*y*+1, −*z*+1; (vi) *x*, −*y*+1/2, *z*+1/2.
